# The Avian Influenza Virus PA Protein Recruits Host RPS27A to Support Viral Replication

**DOI:** 10.3390/v18030317

**Published:** 2026-03-03

**Authors:** Ji Liu, Feihu Guan, Yafen Song, Ye Tian, Jie Zhang, Ling Chen, Aoyang Yan, Haoye Yang, Chenghuai Yang, Qianyi Zhang

**Affiliations:** 1 China Institute of Veterinary Drug Control, Beijing 102600, China; liuji010601@126.com (J.L.); songyafen1@126.com (Y.S.); ty18516952255@163.com (Y.T.); chenl2011521@163.com (L.C.); 15638451297@163.com (H.Y.); 2 College of of Animal Science and Technology, Shihezi University, Shihezi 832003, China; gfh9826@163.com (F.G.); 18236531762@163.com (A.Y.); 3 College of Veterinary Medicine, Gansu Agricultural University, Lanzhou 730070, China; lanmao_515@outlook.com

**Keywords:** avian influenza virus, PA protein, host–virus interactions, multi-omics integration, protein–protein interaction network

## Abstract

Avian influenza, a disease caused by avian influenza virus (AIV), mainly infects birds but can also infect mammals, which poses a serious threat to public health. Therefore, thorough understanding of its pathogenic mechanism and the identification of antiviral targets are essential for the prevention, control, and treatment of AIV. The polymerase acidic protein (PA) is a core component of the viral RNA-dependent RNA polymerase complex and plays a central role in viral transcription through its cap-snatching activity during early infection. We employed a multi-omics approach combining transcriptome analysis with PA interaction proteomics to characterize host responses during AIV infection and explore the PA–host interaction network. Transcriptomics revealed a polarized host response marked by activated translation-related processes, mitochondrial energy metabolism, and innate immune signaling, alongside broad suppression of nuclear transcriptional regulation and cell cycle pathways. Immunoprecipitation–mass spectrometry identified host proteins associated with PA that were enriched in RNA metabolism, ribosome biogenesis, and protein homeostasis. Integrative analysis of transcriptomic and interactome data, along with protein–protein interaction network analysis, prioritized a subset of high-confidence PA-interacting host factors. Among these, ribosomal protein RPS27A was validated to interact with PA and to support viral replication during early infection in this study.

## 1. Introduction

Avian influenza virus (AIV), particularly highly pathogenic avian influenza (HPAI), has continue to circulate widely in global poultry production systems, posing persistent challenges to the poultry industry and biosecurity. The increasing number of cross-species transmission events to mammals highlights potential zoonotic and public health risks [1,2]. So, a detailed understanding of AIV infection and pathogenic mechanisms is crucial for effective prevention and control of avian influenza.

AIV belongs to the genus *Alphainfluenzavirus* within the family *Orthomyxoviridae*. Its genome consists of eight segments of single-stranded, negative-sense RNA. Viral transcription and replication are carried out by the viral RNA-dependent RNA polymerase complex, which consists of the PB2, PB1, and PA subunits [3]. The operates in the host cell nucleus, mediating viral mRNA transcription and genome replication. As a key subunit of the polymerase complex, PA performs two major roles. Its N-terminal endonuclease domain harbors endonuclease activity, which mediates the cleavage step of cap-snatching, thereby generating capped RNA fragments that serve as primers for viral mRNA synthesis [4,5]. PA interacts with PB1 to support the assembly, stability, and activity of the polymerase complex, providing a structural basis essential for efficient viral RNA synthesis additionally [6].

PA has been shown to interact with a range of host factors, allowing influenza virus to adapt to different cellular environments and regulate viral replication and host antiviral responses [7,8,9,10,11,12,13]. Several host proteins have been identified as functional cofactors of PA. ANP32A is essential for influenza virus polymerase activity and plays an important role in species adaptation [14]. CLE/CGI-99 promotes viral genome transcription and support viral RNA synthesis [15]. In addition, the molecular chaperone DNAJA1 enhances viral replication by stabilizing the PA–PB1 polymerase complex [16]. In contrast, certain viral restriction factors also reach their target sites via the PA protein to inhibit viral activity. HDAC6 inhibits PA-mediated polymerase activity through deacetylation [17], whereas IRF3 interacts with the N-terminal domain of PA and suppresses interferon production, thereby modulating innate immune responses [9]. The nuclear transport-associated protein HAX1 blocks PA nuclear localization and limits viral replication [18]. These studies indicate that PA-associated host factors are involved in diverse cellular processes, including RNA synthesis, protein homeostasis, nuclear transport, and immune regulation. However, only a limited number of PA-interacting host proteins have been characterized to date, and the broader host regulatory network through which PA influences cellular functions during infection remains poorly defined. Increasing evidence suggests that ribosomal proteins function beyond ribosome assembly and protein translation and can participate in RNA homeostasis, stress responses, and innate immune regulation. Multiple ribosomal proteins have been reported to be exploited by viruses or to interact with viral proteins, thereby influencing viral replication efficiency [19]. However, the specific roles of ribosomal proteins such as RPS27A during AIV infection, as well as their potential association with viral polymerase proteins, remain poorly defined.

Multi-omics integration has become a useful strategy for studying complex host–virus interactions. Because transcriptomic changes do not always translate directly into protein abundance or functional interactions, combining RNA-seq with proteomics provides complementary evidence to prioritize key host factors and regulatory pathways during viral infection [20,21].

In this study, a PA-centric, multi-omics strategy integrating transcriptomic analysis with interactome-based proteomic screening has been established. Transcriptome sequencing was first performed at the early stage of AIV infection to characterize host transcriptional responses. Subsequently, PA was then used as bait for immunoprecipitation–mass spectrometry to identify candidate interacting proteins. Finally, transcriptional changes, interaction evidence, and protein interaction network analyses were integrated to prioritize key host factors for functional validation. This work defines the PA-associated host interaction network during AIV infection and provides insights into host-dependent pathways that may be targeted for antiviral intervention.

## 2. Materials and Methods

### 2.1. Cells and Virus

Human lung adenocarcinoma epithelial cells (A549) were cultured in Ham’s F-12K (Kaighn’s) medium (Wisent Biotechnology Co., Ltd., Nanjing, China), and human embryonic kidney cell line 293T and the Madin-Darby canine kidney (MDCK) cell line were cultured in Dulbecco’s modified Eagle’s medium (DMEM, Gibco, Grand Island, NY, USA) supplemented with 10% fetal bovine serum (FBS, Gibco, Grand Island, NY, USA) and 1% penicillin–streptomycin (Biosharp, Hefei, China) at 37 °C, 5% CO_2_ in a humidified incubator. All cell lines were kindly provided by the Harbin Veterinary Research Institute, Chinese Academy of Agricultural Sciences. The highly pathogenic H5N1AIV strain A/chicken/HLJ/01/2011 (H5N1) used in this study was identified and preserved by the China Institute of Veterinary Drug Control (IVDC). All virus-related experiments were conducted in a certified Animal Biosafety Level 3 (ABSL-3) laboratory at IVDC in accordance with institutional biosafety regulations.

### 2.2. Transcriptome Sequencing and Analysis

A549 cells were seeded in 10 cm culture dishes and infected with highly pathogenic H5N1 virus at a multiplicity of infection (MOI) of 2. The same amount of phosphate-buffered saline (PBS, pH 7.5) was added to control cells. After 1 h of incubation, cells were washed twice with PBS and maintained in maintenance medium with 2% FBS. Each group was ready in trio biological replications. At 8 h post-infection (hpi), the cells were subsequently harvested, and the total RNA was extracted using TRIzol reagent (Ambion, Thermo Fisher Scientific, Waltham, MA, USA; cat. no. 15596026) according to the manufacturer’s instructions and resubmitted to General Biol (Chuzhou China) to have the transcriptome sequenced.

RNA purity was assessed using a NanoDrop spectrophotometer (Thermo Fisher Scientific, Waltham, MA, USA). Samples with A260/A280 ratios of 1.8–2.1 and A260/A230 ratios >2.0 were considered acceptable. The RNA concentration was further quantified using a Qubit 4.0 Fluorometer (Thermo Fisher Scientific, Waltham, MA, USA). RNA integrity was evaluated prior to library construction. For mRNA library preparation, poly(A)+ mRNA was enriched using oligo(dT) magnetic beads and fragmented prior to cDNA synthesis. First- and second-strand cDNA synthesis, end repair, adaptor ligation, and PCR amplification were performed according to standard protocols. Libraries were sequenced on an Illumina platform using a 2 × 150 bp paired-end configuration. Raw reads were processed using Cutadapt (v1.9.1) to remove adaptor sequences and low-quality bases. Clean reads were evaluated with FastQC (v0.11.9). Reads were aligned to the human reference genome (GRCh38) using HISAT2 (v2.2.1), and gene-level counts were generated using HTSeq (v0.6.1).

Differential expression analysis was conducted using DESeq2 (v1.34.0). *p* values were adjusted using the Benjamini–Hochberg method. Genes with |log_2_ fold change| ≥ 2 and *p* values ≤ 0.05 were defined as differentially expressed genes (DEGs). GOseq was used to conduct Gene Ontology (GO) enrichment analysis conducted across the Biological Process (BP), Cellular Component (CC), and Molecular Function (MF) categories. The significance threshold was over-represented with *p* values ≤ 0.05. Kyoto Encyclopedia of Genes and Genomes (KEGG) pathway enrichment analysis was performed upon a hypergeometric test, and pathways with *p* values ≤ 0.05 were regarded as significant. Visualization was chosen on the top 30 most enriched pathways.

### 2.3. Cloning and Expression of AIV PA Protein

The full-length gene encoding the AIV PA protein was amplified from the A/chicken/HLJ/01/2011 (H5N1) virus strain using PA-specific primers designed based on the PA gene sequence of this strain. The primer sequences were as follows: pcaggs-kpn1-flag-PA-F: GCTCATCGATGCATGGTACATGGAAGACTTTGTGC, pcaggs-Nhe1-flag-PA-R: GTAGTCCATCGATCTGCTAGTTTCAGTGCATGTGCGAGG. The PCR products were purified using a Gel Extraction Kit (Omega Bio-tek, Norcross, GA, USA; cat. no. D2500-02) according to the manufacturer’s instructions. The purified PA fragment was then cloned into the pcaggs expression vector by homologous recombination using the In-Fusion Snap Assembly Master Mix (Takara Bio USA, Mountain View, CA, USA; cat. no. 638948). The recombinant plasmid was transformed into E.coli DH5α competent cells (Takara Bio USA, Mountain View, CA, USA; cat. no. 9057), and positive clones were selected on LB agar plates containing ampicillin. Plasmids were extracted from bacterial cultures using the NucleoBond Xtra plasmid purification kit (Macherey-Nagel, Düren, Germany; cat. no. 740410) and verified by Sanger sequencing. For protein expression, the recombinant pcaggs-flag-PA plasmid was transfected into A549 cells using Lipofectamine 3000 (Thermo Fisher Scientific, Waltham, MA, USA; cat. no. L3000008) according to the manufacturer protocol. Expression of the PA protein was confirmed by Western blot analysis.

### 2.4. Immunoprecipitation–Mass Spectrometry (IP-MS)

A549 cells were transfected with a Flag-tagged PA expression plasmid. Six hours after transfection, the medium was replaced with maintenance medium containing 2% FBS, and cells were cultured for an additional 24 h before harvest. Cells transfected with an empty vector served as controls. Immunoprecipitation was carried out using a Flag-tag Protein IP Assay Kit with Magnetic Beads (Beyotime Biotechnology, Shanghai, China; cat. no. P2181S) according to the manufacturer’s instructions. Briefly, cell lysates were incubated with anti-Flag magnetic beads to enrich Flag-PA and its associated protein complexes. After washing, beads were collected and subjected to Western blot analysis using an DYKDDDDK tag Monoclonal antibody (Proteintech, Wuhan, China; cat. no. 66008-4-Ig), then processed for mass spectrometry analysis by SpecAlly Life Technology Co., (Wuhan, China). Mass spectrometry was performed using an UltiMate 3000 RSLCnano liquid chromatography system coupled with a Q Exactive HF mass spectrometer operating in data-dependent acquisition (DDA) mode. Raw data were analyzed with MaxQuant software (version 1.6.10.43), with the false discovery rate (FDR) for peptide and protein identification controlled at 1%. Label-free quantification was conducted using the MaxLFQ algorithm. Proteins significantly enriched in the Flag-PA group compared with the empty vector control (|log_2_^FC^| ≥ 1, *p* ≤ 0.05) were defined as potential PA-interacting proteins. Functional annotation and pathway enrichment analyses were performed using the GO and KEGG databases.

### 2.5. Protein–Protein Interaction (PPI) Network Analysis

Candidate proteins were submitted to the STRING database (version 11.5), with the species restricted to *Homo sapiens*, to retrieve known and predicted protein–protein interactions. The experimental data, curated databases and text mining obtained provided interaction evidence. PPI was built with minimum interaction confidence (Combined score in STRING) ≥ 0.4, with PPI enrichment *p* values < 0.001. The interaction networks that resulted were then imported in Cytoscape software (version 3.9.1) for visualization and topological analysis. The degree centrality of the networks and network parameters were analyzed using the Network Analyzer plugin. Nodes that were in the top 20 percent regarding the degree of centrality were identified as high-priority candidate host factors to be used in further analysis.

### 2.6. Gene Overexpression and RNA Interference

For gene overexpression, the full-length human RPS27A coding sequence was cloned into the pcaggs expression vector with a Myc tag to generate the pcaggs-Myc-RPS27A plasmid (General Biol, Chuzhou, China). A549 cells were seeded in 6-well plates and transfected at approximately 70–80% confluence with 2.5 μg plasmid DNA per well using Lipofectamine 3000 (Thermo Fisher Scientific, Waltham, MA, USA; cat. no. L3000008) according to the manufacturer’s instructions. Cells transfected with the empty vector were used as controls. Overexpression efficiency was confirmed by Western blot analysis at 24 h post-transfection.

For gene knockdown, small interfering RNAs (siRNAs) targeting RPS27A (RPS27A-siRNA-449: CAGACAUUAUUGUGGCAAATT) and a negative control siRNA were synthesized by General Biol siRNAs were transfected into cells at a final concentration of 50 nM using Lipofectamine 3000 according to the manufacturer’s protocol. Knockdown efficiency was evaluated by RT-qPCR at 24 h post-transfection.

### 2.7. Real-Time Quantitative PCR (RT-qPCR)

A549 cells were infected with H5N1 virus at an MOI of 0.01. Culture supernatants and infected cells were collected at 12, 24, and 48 h post-infection (hpi). Three independent biological replicates were prepared for each group. Viral RNA in culture supernatants was extracted using a Viral DNA/RNA Extraction Kit (BIG FISH, Hangzhou, China; cat. no. BFMP08R) according to the manufacturer’s instructions. Viral NP gene copy numbers were quantified using a One Step RT-qPCR SYBR Green Kit (Vazyme, Nanjing, China; cat. no. Q226) on a real-time PCR system (Vazyme, Nanjing, China). A standard curve was generated using serial tenfold dilutions of an NP gene plasmid with known copy numbers, and absolute viral RNA copy numbers were calculated based on Ct values. For intracellular viral RNA analysis, total RNA was extracted from infected cells using a Total RNA Extraction Kit (BIG FISH, Hangzhou, China; cat. no. BFMP07R) according to the manufacturer’s instructions. Reverse transcription and quantitative PCR were performed using the same RT-qPCR kit. GAPDH was used as an internal control, and relative expression levels were calculated using the 2^−ΔΔCt^ method. Each sample was analyzed in triplicate.

### 2.8. Co-Immunoprecipitation (Co-IP) Analysis

Co-IP experiments were conducted following procedures similar to those described above. The transfection of HEK293T cells was done with Flag-PA plasmid, Myc-RPS27A plasmid or both. At 24 h post-transfection, cells were collected and lysed. Negative controls were completed with empty vectors. The Flag-tag Protein IP Assay Kit with Magnetic Beads was used to purify Flag-PA-associated protein complexes. Western blotting was used to analyze and identify certain interacting proteins.

### 2.9. Western Blot Analysis

Equal amounts of protein were separated by SDS-PAGE and transferred onto PVDF membranes. Membranes were blocked with TBST containing 5% nonfat milk at room temperature for 1 h and then incubated with primary antibodies overnight at 4 °C. After washing three times with TBST (5 min each), membranes were incubated with HRP-conjugated secondary antibodies at room temperature for 1 h. Protein signals were detected using enhanced chemiluminescence (ECL) reagents (Beyotime Biotechnology, Shanghai, China; cat. no. P0018M) and imaged using an Amersham Imager 680 system (Cytiva, Marlborough, MA, USA). GAPDH was used as a loading control.

The primary antibodies used were anti-Myc tag (Proteintech, Wuhan, China; cat. no. 16286-1-AP), anti-Flag tag (Proteintech, Wuhan, China; cat. no. 66008-4-Ig), anti-GAPDH (Proteintech, Wuhan, China; cat. no. 10494-1-AP), anti-RPS27A (Abcam, Cambridge, UK; cat. no. ab172293), anti-AIV NP mouse monoclonal antibody, and anti-AIV PA mouse monoclonal antibody (both kindly provided by the Harbin Veterinary Research Institute). HRP-conjugated goat anti-rabbit IgG (Proteintech, Wuhan, China; cat. no. RGAR001) and HRP-conjugated goat anti-mouse IgG (Proteintech, Wuhan, China; cat. no. RGAM001) were used as secondary antibodies.

### 2.10. Confocal Immunofluorescence Microscopy

HEK293T cells were seeded onto glass-bottom confocal dishes and co-transfected with Flag-PA and Myc-RPS27A plasmids using Lipofectamine 3000 (Thermo Fisher Scientific, Waltham, MA, USA; cat. no. L3000008) according to the manufacturer’s instructions. Cells were transfected at approximately 70–80% confluence with 2.5 μg total plasmid DNA per dish. At 24 h post-transfection, cells were fixed with 4% paraformaldehyde at room temperature for 15 min and washed three times with PBS. Cells were permeabilized with 0.1% Triton X-100 for 10 min, washed again, and blocked with PBS containing 5% bovine serum albumin for 1 h at room temperature. Cells were then incubated with primary antibodies at 4 °C overnight, followed by washing and incubation with fluorescently labeled secondary antibodies for 1 h in the dark. Nuclei were counterstained with DAPI. Images were acquired using a Nikon Eclipse Ti laser scanning confocal microscope (Nikon, Tokyo, Japan) at Wuhan Servicebio Technology Co., Ltd. (Wuhan, China).

### 2.11. Viral Titer Determination and Replication Kinetics

A549 cells were infected with virus at an MOI of 0.01. After 1 h incubation, cells were washed with PBS and maintained in maintenance medium. Culture supernatants were collected at 12, 24, and 48 h post-infection (hpi) for viral titration. Viral titers were determined by plaque assay on MDCK cells. MDCK cells were seeded in 6-well plates and allowed to reach approximately 90% confluence prior to infection. Tenfold serial dilutions of virus-containing supernatants were prepared and added to MDCK cell monolayers for 1 h at 37 °C with gentle rocking. After removal of the inoculum, cells were washed twice with PBS and overlaid with maintenance medium containing 1% agarose and 2% FBS. Plates were incubated at 37 °C for 72 h. Cells were then fixed and stained with crystal violet, and plaques were counted. Viral titers were calculated and expressed as plaque-forming units per milliliter (PFU/mL).

### 2.12. Statistical Analysis

Data visualization and statistical analyses were performed using GraphPad Prism version 10.0. Data are presented as the mean ± standard deviation (SD) of three or more independent experiments unless otherwise indicated. The two groups were compared with each other using Student’s *t*-test. One-way or two-way analysis of variance (ANOVA) was used for comparisons among multiple groups. Statistical significance was defined as *p* < 0.05, with significance levels indicated as *p* < 0.05 (*), *p* < 0.01 (**), *p* < 0.001 (***), and *p* < 0.0001 (****); NS indicates no significant difference.

## 3. Results

### 3.1. Host Transcriptomic Profiling During the Early Stage of H5N1 Highly Pathogenic Avian Influenza Virus Replication

Following cellular entry, AIV viral ribonucleoprotein (vRNP) complexes were delivered into the nucleus, where viral mRNA transcription is initiated. Influenza virus transcription and polymerase subunit accumulation occur mainly during the early phase of infection, with PA expression increasing markedly within the first replication cycle [22,23]. To focus on host transcriptional responses associated with active viral transcription and polymerase function, while decreasing secondary effects of extensive viral replication and cell damage, 8 h post-infection (hpi) was selected for transcriptomic analysis. RNA sequencing was performed to compare infected cells with uninfected controls.

**Figure 1:**
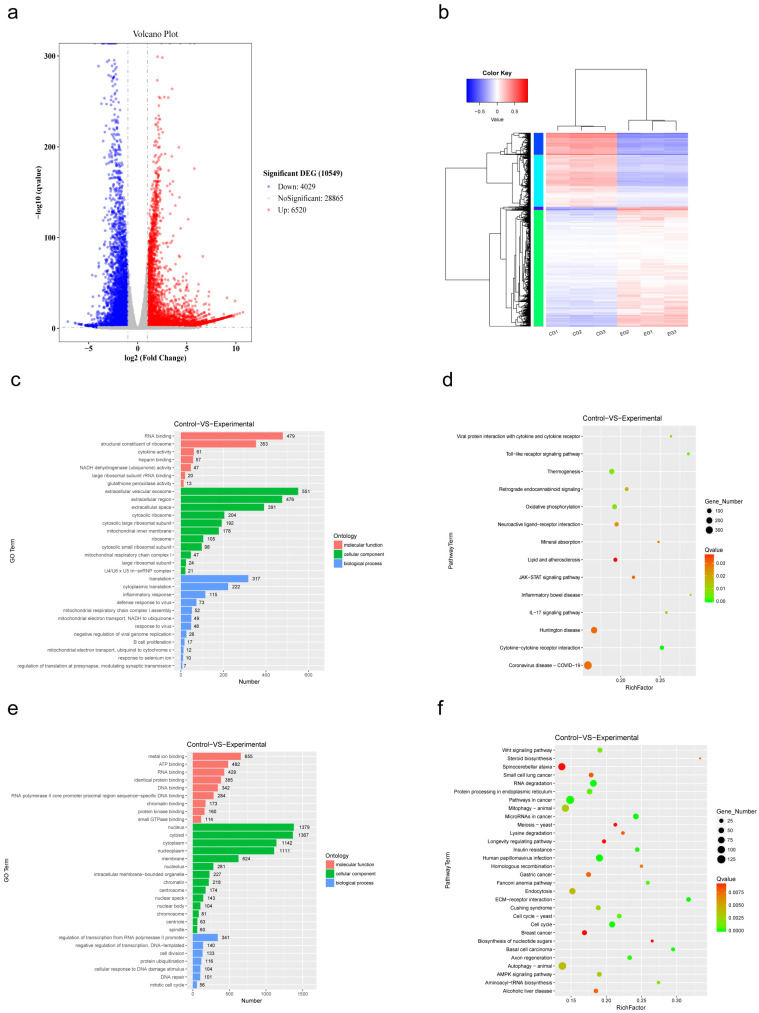
Transcriptomic analysis reveals significant changes in host gene expression following AIV infection. (**a**) Volcano plot showing the differentially expressed genes (DEGs: |log2 fold change| ≥ 2, *p* ≤ 0.05) in A549 cells 8 h post-infection with highly pathogenic avian influenza virus (AIV) compared to controls. Red dots indicate significantly upregulated genes, blue dots indicate significantly downregulated genes, and gray dots indicate genes with no significant change. (**b**) The heatmap of DEG hierarchical clustering. A representative gene forms each row, and a separate sample forms each column. After the normalization, there is red, meaning that it is highly expressed, and blue, meaning that it is low. (**c**) GO enrichment analysis of upregulated DEGs with BP, CC or MF groups. The sizes of bars represent the number of genes that are enriched in a given term. (**d**) KEGG pathway enrichment analysis of DEGs that are upregulated. The number of genes that are considered in every pathway is known as the dot size; color is the statistical significance of enrichment. (**e**) Those are downregulated DEGs in GO analysis. (**f**) Downregulated DEGs in KEGG pathway analysis.

Differential expression analysis identified a total of 10,549 DEGs, including 6520 upregulated and 4029 downregulated genes. AIV infection induced a clear reshaping of the host transcriptional landscape, with upregulated and downregulated genes showing clear separation in both effect size and statistical significance (Figure 1a). Hierarchical clustering further showed strong consistency among biological replicates and a clear separation between infected and control samples (Figure 1b), supporting the reliability of the dataset.

GO enrichment analysis was performed separately for upregulated and downregulated DEGs. At the level of BP, upregulated genes were mainly associated with protein translation, inflammatory responses, and antiviral-related processes (Figure 1c), whereas downregulated genes were enriched in transcriptional regulation, cell division, and protein ubiquitination (Figure 1e). At the CC level, upregulated genes were mainly linked to extracellular regions, exosomes, ribosomes, and the mitochondrial components (Figure 1c). In contrast, downregulated genes were largely associated with nuclear structures (Figure 1e), indicating reduced nuclear activity accompanied by enhanced cytoplasmic and translation-related functions. At the MF level, downregulated genes were strongly enriched in DNA binding, chromatin binding, and RNA polymerase II promoter-specific binding activities (Figure 1e), consistent with suppression of host transcriptional regulation. In addition, molecular functions related to ATP binding, metal ion binding, protein kinase binding, and small GTPase binding were also reduced, suggesting weakened intracellular signaling and regulatory activity.

KEGG pathway analysis was consistent with these findings. Upregulated DEGs were significantly enriched in pathways related to viral infection and innate immune responses, including COVID-19, Toll-like receptor signaling, JAK–STAT signaling, IL-17 signaling, and cytokine–cytokine receptor interaction pathways (Figure 1d). Pathways closely associated with mitochondrial energy metabolism, such as oxidative phosphorylation and thermogenesis, were also significantly enriched. In contrast, KEGG analysis of downregulated genes revealed strong suppression of autophagy-related pathways, including autophagy and mitophagy, as well as several key signaling pathways such as Wnt and AMPK signaling (Figure 1f). These changes suggest that AIV infection broadly inhibits host cell proliferation, genome maintenance mechanisms, and homeostatic regulatory pathways.

Taken together, AIV infection during the transcription–replication stage induces a distinct and polarized host transcriptional response. This response is characterized by enhanced immune activation and energy metabolism, accompanied by widespread suppression of host transcriptional regulation, cell cycle progression, and homeostatic signaling pathways.

### 3.2. Identification of Host Factors Interacting with the PA Protein by IP-MS

**Figure 2:**
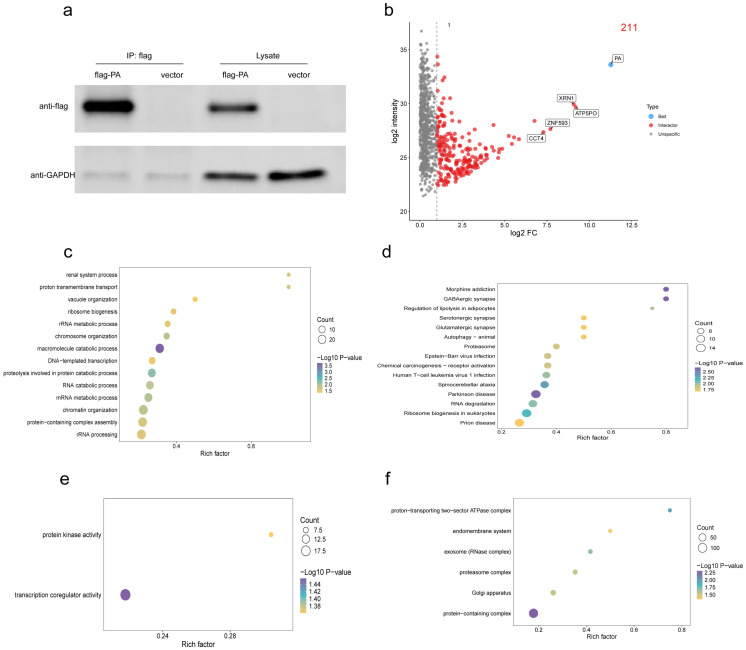
Identification and functional characterization of PA-interacting host proteins. (**a**) Co-immunoprecipitation (IP) of interacting proteins using anti-Flag antibody magnetic beads, followed by Western blot analysis to confirm expression and successful pull-down of Flag-tagged PA. Empty vector was used as negative control, and GAPDH was the internal reference. (**b**) Volcano plot of the protein with a differentially abundant result, comparing flag-PA group with the empty control, highlighted in red as PA-interacting candidate factors (|log2FC| ≥ 1, *p* ≤ 0.05). (**c**,**d**) GO enrichment analysis bubble chart of PA-interacting proteins. (**e**,**f**) KEGG enrichment analysis bubble chart of PA-interacting host proteins.

To further identify host factors that may be directly recruited by the polymerase PA protein to support this functional reprogramming, immunoprecipitation coupled with mass spectrometry (IP-MS) was performed in A549 cells using Flag-tagged PA as bait (Figure 2a). A total of 211 proteins were significantly enriched in the PA immunoprecipitation group when compared with the control group, and 210 host proteins except PA were defined as candidate PA-interacting host proteins (Figure 2b).

To characterize the functional features of these candidate interactors, GO annotation and enrichment analyses were conducted. At the BP level, the enriched proteins were mainly associated with macromolecule catabolic processes, protein degradation, and mRNA metabolism (Figure 2c). At the CC level, these proteins were predominantly localized to protein complexes, proteasome complexes, and exosomes (Figure 2d). At the MF level, significant enrichment was observed in transcriptional co-regulatory activity and protein kinase activity (Figure 2e). Consistent with these results, KEGG pathway analysis revealed significant enrichment of candidate interactors in pathways related to RNA degradation, eukaryotic ribosome biogenesis, the proteasome, and autophagy (Figure 2f).

At the level of RNA regulation, transcriptomic data indicated a functional shift of RNA-binding activities from transcriptional regulation toward translation-related factors during AIV infection. The enrichment of RNA metabolism and RNA quality control factors among PA-interacting proteins further supports a role for PA in shaping an RNA processing environment that favors viral RNA synthesis and replication. In parallel, transcriptional activation of ribosome- and translation-associated pathways was consistent with the enrichment of ribosome biogenesis and protein complex components among PA interactors, suggesting a supportive role of PA within translation-related networks. In addition, although transcriptomic analysis showed a suppression of host autophagy and global protein homeostasis pathways, PA-interacting proteins were significantly enriched in components of the ubiquitin–proteasome system.

Taken together, the integration of transcriptomic and PA interactome data suggests that PA may modulate host functional networks that are extensively reshaped during infection by interacting with host factors involved in RNA metabolism, translational regulation, and protein homeostasis. Through these interactions, PA is likely to contribute to the establishment of a cellular environment that supports viral RNA synthesis and replication.

### 3.3. Integrating Multi-Omics Data to Map PPI Networks Reveals Key PA-Interacting Host Factors

**Figure 3:**
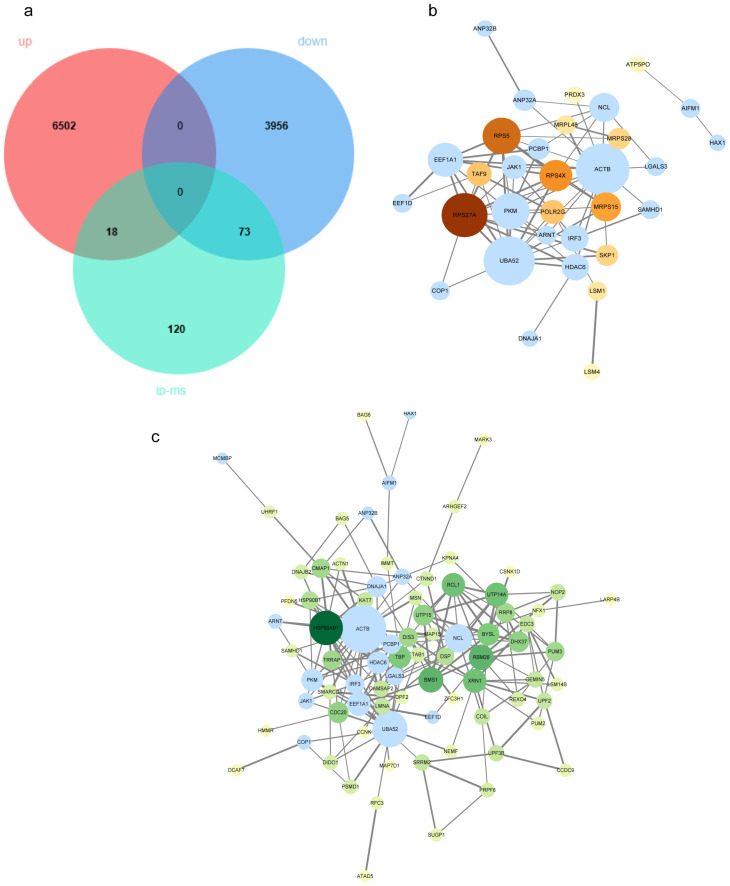
Integrating multi-omics data to map PPI networks. (**a**) A Venn diagram shows the overlap between RNA-seq-identified up- and downregulated DEGs and candidate host factors identified via PA proteomics. (**b**) PPI network based on the overlap between transcriptionally upregulated candidate proteins and experimentally confirmed host factors in protein interaction with influenza A virus (IAV) PA. Centrality in the degree is proportional to the size of the node. Proteins identified as candidate PA-interacting during this experiment appear in orange color, whereas host factors tested before appear in light blue color. (**c**) PPI network based on the intersection of transcriptionally downregulated candidate proteins and already known IAV-PA-interacting host factors. The protein-interacting candidates of PA are in green color.

To increase the confidence and biological relevance of candidate targets, differentially expressed genes (DEGs) identified from transcriptomic analysis were integrated with differentially abundant proteins identified by PA interactome proteomics. Gene identifiers from both datasets were first standardized and mapped to unified gene symbols, and their overlap was visualized using a Venn diagram (Figure 3a). This analysis identified 18 upregulated genes and 73 downregulated genes within the intersecting set (Table S1). Because these candidates were supported by both infection-induced transcriptional changes and potential PA interaction evidence, they were considered a higher-priority pool of host factors.

To further identify key host factors at the network level, the intersecting candidate set was combined with previously reported and experimentally validated AIV host factors from the literature (Table S2). The merged dataset was submitted to the STRING database to construct a PPI network, which was subsequently visualized and analyzed using Cytosccape. Nodes ranked in the top 20% by degree were defined as high-priority host factors, identifying nine high-confidence candidate factors (Table 1). Among these high-confidence factors, all transcription-upregulated factors belonged to ribosomal proteins, including RPS27A, RPS5, RPS4X, and MRPS15. Notably, RPS27A exhibited high connectivity within the network and occupied a central position linking translation-related components to the ubiquitin–proteasome system (Figure 3b). In contrast, the highly confident downregulated factors were primarily associated with functional modules related to chaperone-mediated protein homeostasis, ribosomal biosynthesis, RNA quality control, and transcription and cell cycle regulation. These included HSP90AB1, BMS1, RBM28, UTP14A, RCL1, XRN1, TBP, BYSL, CDC20, UTP15, and DHX37. HSP90AB1 exhibits relatively high connectivity within the network, suggesting that these chaperones may participate in viral protein folding, maintenance of replicative complex stability, and regulation of host signaling pathways during infection (Figure 3c).

### 3.4. AIV Replication Modulated by RPS27A Interacting with PA Protein

RPS27A encodes a ribosomal protein that is essential for ribosome biogenesis and protein translation [24]. RPS27A has also been reported to exert extraribosomal functions, including regulation of apoptosis and cell cycle processes [25]. Previous studies further indicate that multiple ribosomal proteins can interact with viral mRNAs or viral proteins, thereby contributing to viral protein synthesis and regulation of viral replication [19,26].

**Figure 4:**
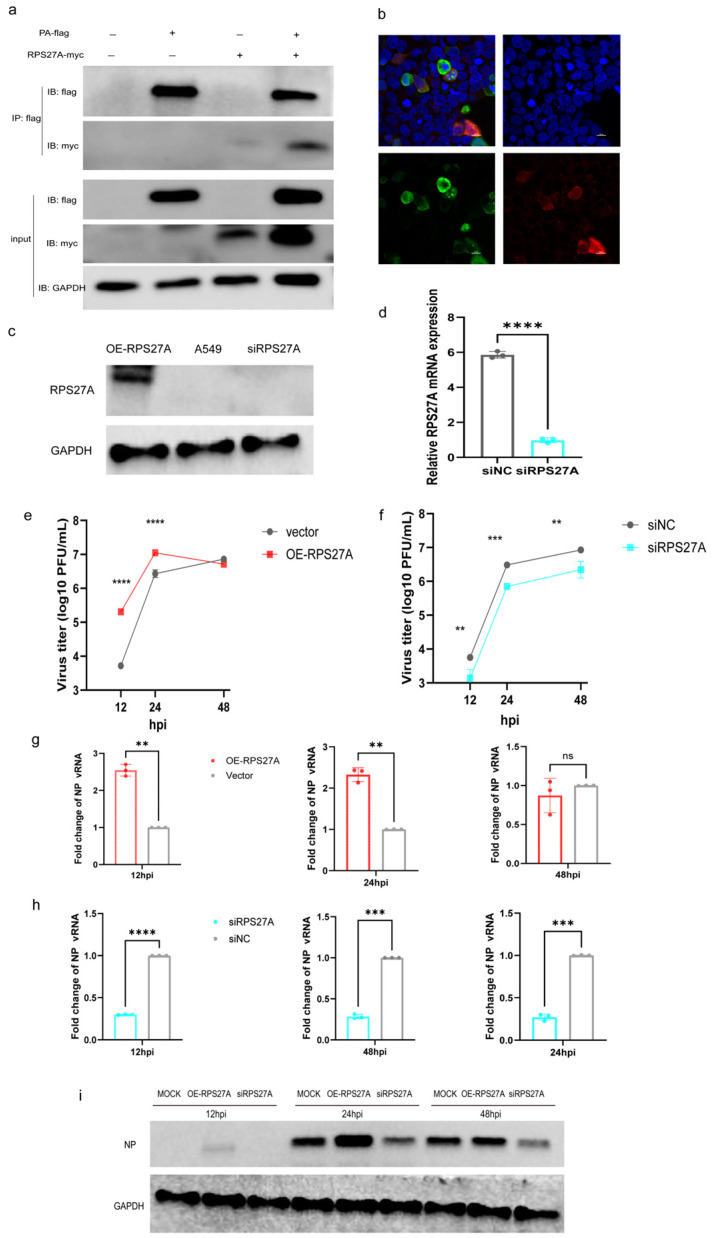
RPS27A interacts with PA protein to promote avian influenza virus replication. (**a**) Co-immunoprecipitation (co-IP) was performed in HEK293T cells after transfecting Flag PA plasmid alone or co-transfecting it with Myc-RPS27A plasmid. (**b**) Confocal immunofluorescence microscopy of cells co-transfected with PA and RPS27A expression plasmids. In this panel, green fluorescence indicates PA (lower left), red fluorescence indicates RPS27A (lower right), and blue fluorescence indicates nuclei (upper right). The merged image (upper left) shows partial co-localization of PA and RPS27A in the cytoplasm. (**c**) Western blot analysis of RPS27A protein expression levels in A549 cells overexpressing RPS27A. (**d**) qPCR detection of relative RPS27A mRNA expression in A549 cells transfected with siRPS27A after 48 h. (**e**–**i**) A549 cells overexpressing or interfering with RPS27A were infected with AIV at MOI = 0.01. Cell supernatants and lysates were collected at 12, 24, and 48 h. Plaque assays determined viral titers in supernatants, RT-qPCR quantified viral NP vRNA levels, and WB analyzed viral NP protein expression in lysates. Data are presented as mean ± standard deviation: ** *p* < 0.01, *** *p* < 0.001, **** *p* < 0.0001, ns, not significant.

To validate the interaction between RPS27A and the AIV PA protein, Flag-tagged PA and Myc-tagged RPS27A expression plasmids were co-transfected into HEK293T cells, followed by co-immunoprecipitation analysis. Myc-RPS27A was readily detected in Flag immunoprecipitates, while no specific signal was observed in cells transfected with control plasmids alone (Figure 4a), indicating a specific intracellular interaction between RPS27A and PA. The spatial distribution of these two proteins was further examined by immunofluorescence staining and confocal microscopy. PA and RPS27A exhibited distinct colocalization signals in the cytoplasmic region (Figure 4b), supporting the possibility of their intracellular complex formation.

To further evaluate RPS27A’s impact on AIV replication, we overexpressed and interfered with the RPS27A gene in A549 cells. Western blot analysis confirmed successful overexpression of the RPS27A protein in A549 cells (Figure 4c). However, endogenous RPS27A was undetectable in the blank group via WB. Therefore, qPCR was employed to assess mRNA expression levels, revealing significantly lower RPS27A mRNA levels in the interference group compared to the control group (Figure 4d).

Cells overexpressing RPS27A were infected with an MOI of 0.01, and viral replication levels were assessed at 12, 24, and 48 hpi. Compared to the empty vector control, RPS27A overexpression significantly enhanced early viral accumulation, with markedly higher viral titers observed at 12 hpi and 24 hpi. This difference was no longer apparent at 48 hpi (Figure 4e). Consistent with these findings, quantitative PCR analysis revealed a similar trend in viral Nucleoprotein (NP) gene copy numbers (Figure 4g), and Western blot analysis further confirmed increased NP protein expression at early time points (Figure 4i)

In contrast, AIV infection experiments in cell lines with RPS27A expression interfered by siRNA demonstrated a marked suppression of viral replication. Compared to control cells, both viral titers and NP gene copy numbers were significantly reduced at 12–48 hpi (Figure 4f,h,i). These findings indicate that RPS27A actively regulates AIV replication at the cellular level, and alterations in RPS27A expression directly impact viral replication efficiency.

## 4. Discussion

To replicate efficiently, viruses must remodel the host cell by commandeering its molecular machinery, a process driven by dynamic interactions between viral and host proteins [27,28]. In this study, we focused on the PA protein of HPAIV and employed an integrated multi-omics strategy to systematically map PA-associated host functional networks during early infection. Our analysis revealed extensive reprogramming of host pathways, with a pronounced upregulation of translation-related processes. Among the altered host factors, the ribosomal protein RPS27A emerged as a high-confidence PA-interacting candidate. Subsequent functional validation confirmed that RPS27A directly interacts with PA and acts as a proviral host factor, significantly enhancing AIV replication.

This study demonstrates that during the early stages of AIV infection, host translation-related processes are significantly activated, including enhanced ribosomal structural component and RNA-binding activities. This phenomenon aligns with the fundamental characteristic of viral replication being highly dependent on the host protein translation system [29]. Previous research indicates that influenza virus infection can induce alterations in ribosome-associated protein abundance and translational activity, thereby enhancing the translation efficiency of viral mRNA [19]. In our subsequent integrated data analysis screening for high-confidence PA-associated host factors, we also identified multiple ribosomal proteins that were significantly upregulated at the transcriptional level and occupied central positions within the interaction network. Consistent with our findings, previous mass spectrometry-based proteomic studies have also reported an increased abundance of ribosomal proteins in host cells following influenza A virus infection [30]. Viruses rely heavily on the host protein translation system to complete their life cycles, while the role of host ribosomal proteins in viral transcription, replication, and antiviral immune responses has gradually gained attention [19,27]. In certain viral infection models, ribosomal proteins may even be “hijacked” by viruses, directly participating in the regulation of viral protein translation and replication processes [31].

We identified multiple potential PA-interacting host proteins through multi-omics analysis. Among these candidate factors, RPS27A was prioritized for subsequent investigation. The PPI network constructed by integrating multi-omics data revealed that RPS27A exhibited a high degree value, positioning it at the core of the network. In independent IP-MS screening results, RPS27A also demonstrated strong interaction signals. Network analysis further revealed that RPS27A forms relatively concentrated interaction modules with proteins including RPS5, RPS4X, UTP14A, RCL1, BYSL, and BMS1 (Figure S1). These proteins are primarily involved in ribosome biogenesis, rRNA processing, and translational regulation. RPS27A is synthesized as a ubiquitin fusion protein and plays an important role in ribosome biogenesis and protein synthesis [32]. We confirmed a direct physical interaction between PA and RPS27A, providing a mechanistic link between the viral polymerase and the host translation machinery. Functional assays further demonstrated that RPS27A overexpression boosted early-stage viral replication, whereas its knockdown exerted a sustained inhibitory effect, establishing its role as a positive regulator of the HPAIV life cycle. It should also be noted that other host factors associated with PA in this study are involved in processes such as translational regulation, RNA processing, or cell cycle regulation, and may also play roles in viral replication. Their specific functions remain to be further investigated.

The significance of RPS27A extends beyond influenza. RPS27A has also been reported to interact with African swine fever virus pCP312R to suppress host protein translation and promote viral replication [33], and has been identified as a key host factor involved in SARS-CoV-2 replication [34]. These observations suggest that RPS27A may serve conserved functions across different viral infections.

We also found that RPS27A expression was higher in the co-expression group of PA and RPS27A compared to the RPS27A-only group (Figure 4a). Consistent with our findings, influenza virus infection upregulates RPS27A transcription (Figure S2). PA overexpression may partially mimic viral infection conditions and activate host cell response pathways, thereby promoting RPS27A expression. Alternatively, direct interaction with PA could enhance RPS27A protein stability. Further investigation is needed to clarify the underlying mechanisms. Intriguingly, RPS27A knockdown was associated with heightened interferon and interferon-stimulated gene (ISG) expression, implying a potential role in modulating host innate immunity (Figure S3). Although several ribosomal proteins have been implicated in innate immune regulation [35], direct evidence linking RPS27A to immune modulation during influenza virus infection remains limited. This potential role warrants further mechanistic investigation.

## 5. Conclusions

In this study, we integrated RNA-seq and PA-bait IP-MS to systematically characterize early host responses and PA-associated interaction networks during highly pathogenic AIV infection. We identified a polarized early host response favoring translation and energy metabolism, and through systematic prioritization, validated RPS27A as a novel PA-interacting proviral factor. These findings advance our understanding of influenza virus pathogenesis by revealing how the viral polymerase directly engages the host translational machinery. Furthermore, the conserved nature of RPS27A exploitation across viruses highlights its potential as a target for developing host-directed, broad-spectrum antiviral strategies.

## Figures and Tables

**Table 1:** Nine high-confidence host factors associated with PA.

Host Actors	Gene Function Descriptions
RPS27A	Component of the 40S ribosomal subunit, involved in protein synthesis and ribosome assembly.
RPS5	Structural constituent of the 40S ribosomal subunit, essential for translation initiation.
RPS4X	Ribosomal protein of the 40S subunit, involved in mRNA translation and ribosome function.
MRPS15	Mitochondrial ribosomal protein required for mitochondrial protein synthesis.
HSP90AB1	Molecular chaperone involved in protein folding, stabilization, and stress response.
BMS1	GTP-binding protein essential for 18S rRNA processing and small ribosomal subunit biogenesis.
RBM28	RNA-binding protein involved in ribosome biogenesis and pre-rRNA processing.
UTP14A	Component of the small subunit processome, required for 18S rRNA maturation.
RCL1	Endoribonuclease involved in pre-rRNA processing during ribosome biogenesis.
XRN1	5′–3′ exoribonuclease involved in mRNA degradation and RNA quality control.
TBP	TATA-box binding protein required for transcription initiation by RNA polymerase II.
BYSL	Nucleolar protein involved in ribosome biogenesis and cell proliferation.
CDC20	Cell cycle regulator that activates the anaphase-promoting complex/cyclosome (APC/C).
UTP15	Component of the small subunit processome, required for ribosomal RNA processing.
DHX37	RNA helicase involved in ribosome biogenesis and pre-rRNA processing.

## Data Availability

The data used to support the findings of this study are available from the corresponding author upon request.

## Supplementary Materials

The following supporting information can be downloaded at https://www.mdpi.com/article/10.3390/v18030317/s1, Table S1: Genes identified at the intersection of transcriptomes and protein interactomes. Table S2: Host factors associated with influenza PA function reported in the literature. Figure S1: PPI network of key PA-interacting proteins identified by IP-MS. Figure S2: Upregulation of RPS27A mRNA expression following H5N1 infection. Figure S3: Interferons and interferon-stimulated gene (ISG) expression in cells targeting RPS27A.

## Abbreviations

The following abbreviations are used in this manuscript:

AIV	Avian influenza virus
ABSL-3	Animal Biosafety Level 3
DEGs	Differentially expressed genes
HPAI	Highly pathogenic avian influenza
IP-MS	Immunoprecipitation–mass spectrometry
MOI	Multiplicity of infection
NP	Nucleoprotein
PA	Polymerase acidic protein
PFU	Plaque-forming unit
PPI	Protein–protein interaction
RT-qPCR	Reverse transcription quantitative real-time PCR
vRNP	Viral ribonucleoprotein
