# Supplementary material for: The Avian Influenza Virus PA Protein Recruits Host RPS27A to Support Viral Replication
Source: Viruses. 2026 Mar 3;18(3):317. doi: 10.3390/v18030317 (PMC13030293; doi:10.3390/v18030317)
Supplement: Supplementary file 1 [file viruses-18-00317-s001.zip › Figure S3.pdf]

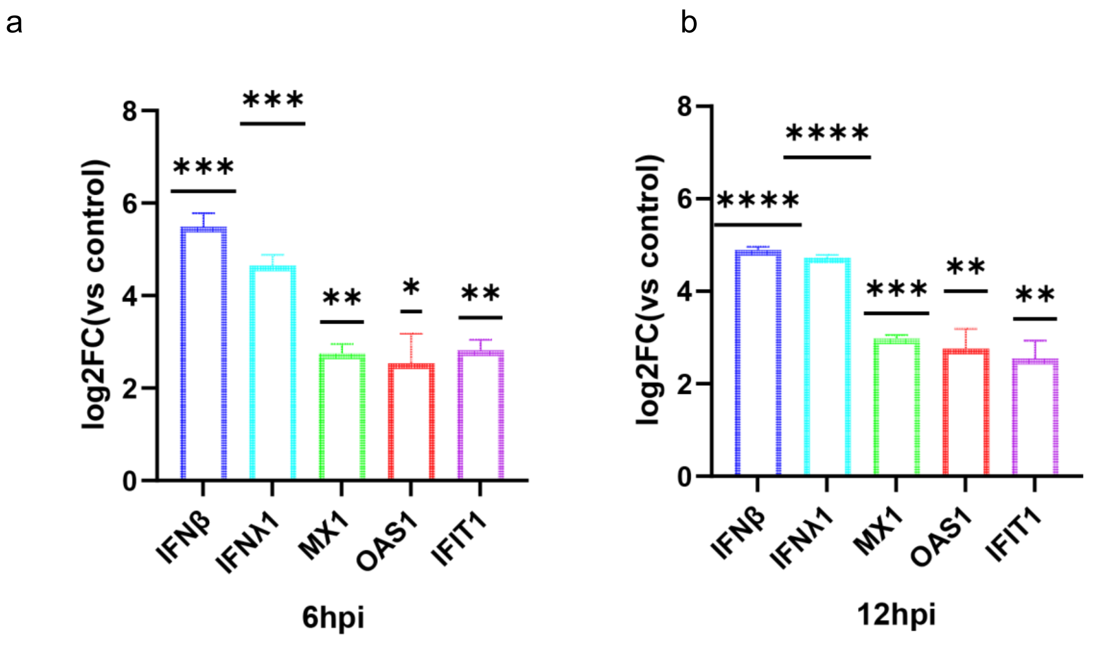

Figure S1. Interferons and interferon-stimulated gene (ISGs) expression in interferon-stimulated cells targeting RPS27A. (a)(b) A549 cells targeting RPS27A gene interference (control group transfected with siNC) were infected with AIV with 2MOI. Collect total RNA from cells at 6 hpi and 12 hpi, and detect the transcription of interferon and ISGs (MX1, OAS1, IFIT1) using qPCR. The relative expression levels of interferons and ISGs mRNA in the RPS27A interference group were significantly higher than those in the control group at 6h and 12h. Data are presented as mean ± standard deviation: **P<0.01, ***P<0.001, ****P<0.0001, NS, not significant.
