# Supplementary material for: The Avian Influenza Virus PA Protein Recruits Host RPS27A to Support Viral Replication
Source: Viruses. 2026 Mar 3;18(3):317. doi: 10.3390/v18030317 (PMC13030293; doi:10.3390/v18030317)
Supplement: Supplementary file 1 [file viruses-18-00317-s001.zip › Figure S1.pdf]

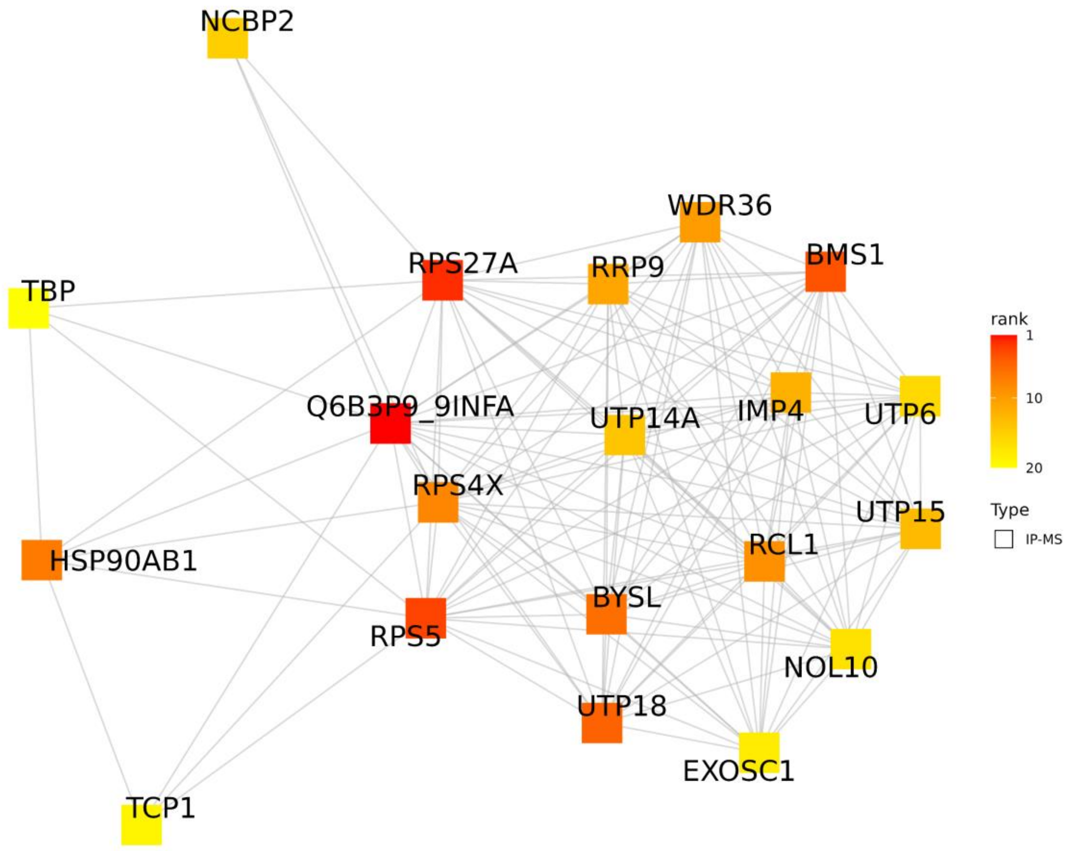

Figure S1. PPI network of key PA-interacting proteins identified by IP-MS. The network was constructed using the STRING database and visualized in Cytoscape. Node color indicates degree ranking, with darker colors representing higher connectivity. Edges represent predicted or experimentally validated interactions.
