# Supplementary material for: The Avian Influenza Virus PA Protein Recruits Host RPS27A to Support Viral Replication
Source: Viruses. 2026 Mar 3;18(3):317. doi: 10.3390/v18030317 (PMC13030293; doi:10.3390/v18030317)
Supplement: Supplementary file 1 [file viruses-18-00317-s001.zip › Figure S2.pdf]

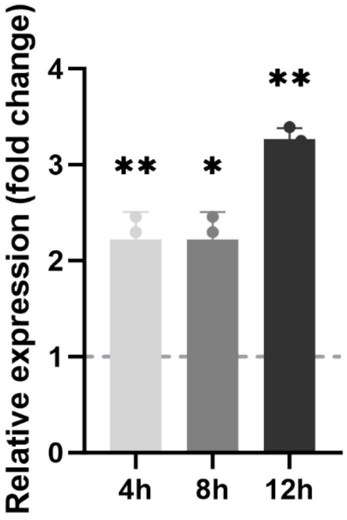

Figure S2. Upregulation of RPS27A mRNA expression following H5N1 infection. RPS27A mRNA expression in A549 cells following H5N1 infection. Cells were infected at an MOI of 0.01 and total RNA was collected at the indicated time points. Relative expression levels were determined by RT-qPCR using GAPDH as an internal control and calculated using the $2^{-\Delta\Delta Ct}$ method. Data are presented as mean ± SD (n = 3). *P < 0.05, **P < 0.01 versus mock control.
